# Design of Micro- and Nanoparticles: Self-Assembly and Application

**DOI:** 10.3390/nano12030430

**Published:** 2022-01-27

**Authors:** Pavel Padnya, Ivan Stoikov

**Affiliations:** A.M. Butlerov Chemistry Institute, Kazan Federal University, 420008 Kazan, Russia

The modern world throws down an increasing number of challenges to humanity. The development of nanotechnology and the creation of nanomaterials of the future are possible solution to the emerging threats. Today, nanomaterials are in demand in the most pressing research areas starting from solving problems with diagnosing COVID-19 and developing antiviral drugs and finishing with new energy sources and environmental challenges. 

The use of self-assembly of organic or/and inorganic components to create the nanomaterials of the future is a promising area of research for many scientists due to the limitless possibilities of designing various architectures for many tasks of modern technologies [1]. This Special Issue covers a wide range of research topics related to the design and application of nanomaterials, including micro- and nanoparticles based on self-assembly. 

The development of new approaches to the synthesis of such nanomaterials is urgent due to the promising prospects of the resulting nanostructures with a broad application potential. The use of nanoparticles in biomedicine offers new opportunities for the development of drug delivery systems [2]. Nakamura et al. has proposed a novel protein carrier system capable of delivering various proteins to cells in vitro and in vivo [3]. The uniqueness of this system is in its easy two-step method of self-assembling of protamine, low molecular heparin, and cell penetrating peptides. The obtained system has great potential for use, especially in the field of animal biotechnology and biomedicine.

The authors of the article [4] have developed a pH-sensitive supramolecular nanosystem based on decasubstituted pillar[5]arenes and tetrazole-containing polymers. A series of pillar[5]arene derivatives containing tosylate, phthalimide, and primary and tertiary amino groups was synthesized to create novel pH-sensitive nanosystems. It was shown that nanoparticles, stable at neutral pH and degraded in acidic medium (pH = 5), were formed by the interaction of the synthesized pillar[5]arenes with tetrazole-containing polymers. The results obtained are a step toward the creation of new universal stimulus-sensitive drug delivery systems.

Due to the active influence of viral diseases, it is important to search for new antiviral agents, as well as to establish the mechanism and potential of existing medicines. Severe acute respiratory syndrome (SARS), Middle East respiratory syndrome (MERS), and coronavirus disease (COVID-19) are the most well-known diseases affecting the life quality of the population. The prospect of using nanomaterials and nanoparticles (silver, gold, quantum dots, organic nanoparticles, liposomes, dendrimers, and polymers) against coronaviruses was discussed in the review [5]. The results obtained during the discussion can be actively used for the development of relevant antiviral drugs against coronaviruses and other viral diseases. 

The use of various biomolecules in modern bioengineering and nanotechnology makes it possible to create new nanomaterials with attractive properties such as biocompatibility, biodegradability, the ability to reproduce natural cellular microenvironment, and minimal toxicity. A recent review [6] brings together publications on the design, construction, and in vitro and in vivo research of multilayer capsules and nanoparticles based on biopolymers mimicked on non-porous and porous particles and crystalline drugs. Based on the analysis and summary of the results obtained for many years, the authors conclude about the prospects and problems of this topic. 

Environmental pollution by industry and non-ecological modes of transport is another problem of the 21st century. The transfer to electric vehicles can solve it. However, the problem of creating modern storage batteries has appeared in this way. LiFePO_4_, often used as a cathode, is one of the promising materials in lithium-ion batteries. Ruiz-Jorge et al. [7] investigated the effect of the heating rate on the hydrothermal synthesis of LiFePO_4_ particles. It turned out that it is important to provide low heating rates in the temperature range of 130–150 °C for the formation of large amounts of LiFePO_4_ particles with good levels of crystallinity. The results obtained open up possibilities for the rapid industrial synthesis of the materials required for storage batteries.

When creating micro- and nanoparticles, it is important to learn how to control the synthesis process, not only for the reproducibility of the results obtained but also in order to search for the possibility of predicting the properties of the target nanostructures [8]. Prof. Duguet and co-workers [9] obtained submicron polystyrene particles with several 100 nm silica or magnetic silica patches emerging at their surface by self-assembly under the solvent action. Thus, the authors were able to develop a method for obtaining and controlling the morphology of polymeric multipatch particles. Further use of the obtained microparticles with inorganic patches was expected in various applications as nanomotors, nanostirrers, nanoswimmers, e-paper pixels, targeted drug delivery, and water treatment. 

Prof. Toprak’s scientific group suggested another example of the controlled synthesis of the nanoparticles [10]. The effect of the addition of a series of salts and surfactant additives (poly(vinyl pyrrolidone), sodium acetate, sodium citrate, hexadecyltrimethylammonium bromide, hexadecyltrimethylammonium chloride, and potassium bromide) on the morphology of Rh nanoparticles was investigated. The correlation between the use of the studied additives and the shape of rhodium nanoparticles (trigonal, cubic, or spherical ones) was shown. The authors studied the cytotoxicity of the obtained Rh nanoparticles and assessed the cell lines of macrophages and ovarian cancer. The obtained results are important and can be useful for the design of the Rh-nanoparticles-based contrast agents for X-ray fluorescence computed tomography bio-imaging.

Supramolecular self-assembly is a promising way to obtain functional nanosystems [11]. The use of synthetic macrocyclic compounds such as crown ethers, cyclodextrins, cucurbiturils, calixarenes, porphyrins, and pillararenes opens up wide possibilities for creating such systems [12,13,14,15]. New amphiphilic calix[4]arene derivatives containing *N*-alkyl/aryl imidazolium/benzimidazolium fragments capable of self-assembly into the submicron nanoparticles in water were synthesized [16]. The addition of the obtained compounds to multilayer 1,2-dipalmitoyl-sn-glycero-3-phosphocholine (DPPS) vesicles resulted in the formation of single-layer calixarene/DPPS vesicles, which are capable of enhancing the catalytic activity in Suzuki–Miyaura coupling.

Organic dyes and their nanosized polymeric forms are widely used in the design of electrochemical sensors based on the self-assembly of organic components [17]. A novel DNA sensor for the determination of doxorubicin has been developed by the consecutive electropolymerization of an equimolar mixture of organic dyes (azure B and proflavine) and the adsorption of the DNA from salmon sperm on the polymer film [18]. The developed electrochemical biosensor can detect the anticancer drug doxorubicin in the concentration range from 0.03 to 10 nM with the limit of detection of 0.01 nM. The DNA sensor was tested on doxorubicin preparations and on spiked samples of artificial blood serum. It can be used for the determination of drug residues in blood and for pharmacokinetic studies.

A cerium oxide (CeO_2_) nanostructures possess unique catalytic and redox properties, comprehensively described in the review [19]. Ceria’s nanomorphology is an important factor affecting its reactivity. Therefore, the development of new methods for obtaining these nanomaterials with different shapes based on various biotemplates is actual. The authors analyzed the relationship between the nanomorphology of nanocerium and its reactivity. They described biotemplates used in recent years for the synthesis of cerium nanostructures and their (bio)applications in many areas such as catalysis for energy consumption, environmental preservation, and remediation.

Due to the active development of new synthetic methods for nanomaterials, the influence of impurities formed in the synthesis are challenges to the quality of the obtained nanostructures. The search for the methods of their purification is an inalienable part of the nanoparticles’ design. Prof. Prior and co-workers, in their review article [20], summarized the latest advances in silver nanoparticle purification techniques. The authors identified several commonly used approaches, such as magnetic and hydrodynamic force-based methods, chromatography, density gradient centrifugation, electrophoresis, selective precipitation, membrane filtration, and liquid extraction. Due to the possible toxicity of organic and/or inorganic impurities, this review is relevant for many researchers planning their work in the nanotechnology field, especially in case of biological applications of the obtained nanomaterials.

Two-dimensional nanomaterials are another interesting type of nanomaterials. There are two key approaches of obtaining 2D nanomaterials, namely, top-down and bottom-up technologies [21]. Each of them has its own advantages and disadvantages [22]. The production of nanolayers using top-down approaches, such as the layering of bulk layered oxides, is one of the most promising directions in the field of 2D nanomaterials. Using the top-down approach, Silyukov and co-workers [23] showed the formation of stable suspensions of perovskite nanolayers and their coatings. They were obtained by exfoliation of the protonated bismuth titanate and its *N*-alkylamine derivatives in an aqueous solution of tetrabutylammonium hydroxide. The resulting nanolayers and coatings can be used as functional materials for catalysis and electronics.

Self-assembling in solid state 2D nanosheets based on thiacalix[4]arene derivatives functionalized by geranyl fragments at the lower rim in *cone* and *1,3-alternate* conformations were obtained by the bottom-up approach [24]. The choice of terpenoid macrocyclic derivatives was explained by their ability to polymorphism and crystalline modifications, as well as non-toxicity. The obtained 2D nanomaterials can be used for the creation of high-energy materials and polymorphic structures for pharmaceuticals.

Overall, this Special Issue includes 13 excellent papers of the contributors from 10 countries in the design and application of nanomaterials, including micro- and nanoparticles, and covers various fields of nanotechnology, chemistry, biology, and material science. As guest editors of the Special Issue “Design of Micro- and Nanoparticles: Self-Assembly and Application”, we thank all authors and reviewers for their valuable contributions. We hope that the publications presented in this Special Issue will attract even greater interest from scientists around the world.
